# Single‐cell transcriptional profiling of human carotid plaques reveals a subpopulation of endothelial cells associated with stroke incidences

**DOI:** 10.1111/jcmm.17354

**Published:** 2022-05-08

**Authors:** Fengchan Li, Yun Du, Lei Hong, Ziting Liu, Kunmin Yan, Chu Liu, Zhen Zhu, Qiongyu Lu, Chaojun Tang, Li Zhu

**Affiliations:** ^1^ 12582 Cyrus Tang Hematology Center Collaborative Innovation Center of Hematology Suzhou Key Laboratory of thrombosis and vascular diseases Soochow University Suzhou China; ^2^ Department of The First Affiliated Hospital of USTC Anhui China; ^3^ National Clinical Research Center for Hematologic Diseases of the First Affiliated Hospital of Soochow University Suzhou China

**Keywords:** cellular heterogeneity, endothelial cells, human carotid artery plaque, scRNA‐seq

## Abstract

The differences in plaque histology between symptomatic and asymptomatic patients have been widely accepted. Whether there is a heterogeneity of cells between symptomatic and asymptomatic plaques remains largely unclear. To reveal the potential heterogeneity within different plaques, which may contribute to different stroke incidences, we obtained the scRNA‐seq data from symptomatic and asymptomatic patients and identified eight cell types present in plaques. Further analysis of endothelial cells (ECs) revealed three distinct EC subpopulations appeared to be endowed with specific biological functions such as antigen processing and presentation, cell adhesion, and smooth muscle cell proliferation. Of note, the differentially expressed genes of the EC 2 subpopulation showed that the genes involved in cell adhesion were up‐regulated in asymptomatic plaques compared to symptomatic plaques. Integrating the data of intraplaque haemorrhage and plaque stability, the 5th top‐enriched biological process was cell adhesion in the stable or non‐haemorrhaged plaques compared to unstable plaques or haemorrhaged plaques. Among these cell adhesion‐related genes, the intersection gene AOC3 may play a vital role in plaque haemorrhage and plaque stability. Targeting cell adhesion and the specialized genes may provide potential new therapeutic directions to prevent asymptomatic patients from stroke.

## 1 INTRODUCTION

Carotid atherosclerosis is a complexly chronic inflammatory disease that can lead to symptoms such as stroke.^1^ An ischaemic stroke may occur as a result of the rupture of unstable atherosclerotic plaque.^2^ Unstable plaque material or fresh thrombus travels into the brain causing an ischaemic event by occluding a cerebral artery in the symptomatic patient, whereas plaques may remain stable and patients asymptomatic for decades.^3^ However, only a small proportion of asymptomatic patients with a significant carotid artery stenosis will develop a stroke or transient ischaemic attack.^4^ Thus, we speculated that the diverse cells in the carotid artery plaque may have contributed to these different clinic outcomes. Importantly, a single‐cell RNA‐seq study revealed that plaques from symptomatic patients were characterized by a distinct subset of CD4+ T cells and by T cells that were activated and differentiated, while T cells and macrophages of plaques from asymptomatic patients were activated and displayed evidence of IL‐1β signalling.^5^ However, it is still unclear whether the difference in the heterogeneity of endothelial cells between symptomatic and asymptomatic plaques leads to different stroke incidences.

Functional heterogeneity of endothelial cells has been characterized in single tissues^6^, ^7^, ^8^, ^9^, ^10^, ^11^ and multiple tissues^12^, ^13^, ^14^ using scRNA‐seq technology. For multiple tissues, Kalucka J, et al.^12^ showed that the tissue type contributed to the EC heterogeneity while ECs from different vascular beds exhibited transcriptome similarity across tissues. For single tissues, atherosclerotic ECs are also highly heterogeneous and plastic both in mouse plaques^9^ and human plaques.^10^ EC subpopulation of EMT‐related cells are increased during the development of atherosclerosis, which highly expresses mesenchymal and extracellular matrix genes, such as Tgfbr2, Fn1, Eln, Vim, Dcn and Mgp.^10^ In line with the findings in mice, a subpopulation of human atherosclerotic ECs expresses typical SMC markers indicating that this subset may be undergoing endothelial to mesenchymal transition (EMT).^10^ In addition, other heterogeneous EC subpopulations show distinct expression of ACKR1 involved in angiogenesis and regeneration of damaged endothelium.^10^

To discover the potential heterogeneity of the ECs in asymptomatic and symptomatic human plaques, we integrated the scRNA‐seq data from symptomatic and asymptomatic human carotid artery plaques. Eight cell types including SMCs, fibrochondrocytes, macrophage, SMC‐derived intermediate cells, ECs, T cells, fibroblasts and mast cells were singled out using Seurat‐based cell clusters. Among the endothelial cells, three distinct EC subpopulations with unique gene signatures were identified based on the unsupervised Seurat‐based clustering. Gene ontology term analyses revealed that EC 1 cluster was endowed with specific functions such as angiogenesis, EC 2 cluster showed its particular functions in cell adhesion, while EC 3 cluster displayed enrichment of specialized functions such as positive regulation of smooth muscle cell proliferation. Of note, we identified 240 differentially expressed genes (DEGs) in the EC 2 subpopulation of asymptomatic group compared to symptomatic group. Top 5 biological processes such as regulation of glucose metabolic process, cell adhesion, negative regulation of canonical Wnt signalling pathway, triglyceride catabolic process and cell‐matrix adhesion were up‐regulated in EC 2 subcluster of asymptomatic group. Due to the symptomatic plaques tend to be unstable and prone to rupture, we integrated the data of intraplaque haemorrhage and plaque stability and found that the biological function of cell adhesion was up‐regulated in stable and non‐intraplaque haemorrhage plaques. Further analysis revealed that the intersection gene AOC3 involving cell adhesion may be regulated by the PI3K‐Akt signalling pathway through ITGA6 and may play a vital role in plaque haemorrhage and plaque stability.

## 2 MATERIALS AND METHODS

### 2.1 Data collection and preprocessing

The gene expression profiles of GSE155512, GSE163154 and GSE120521 were downloaded from the Gene Expression Omnibus (GEO). GSE155512 is a single‐cell transcriptome file of human atherosclerotic carotid arteries obtained from three patients including 1 symptomatic patient and 2 asymptomatic patients.^15^ The platform GPL24676 Illumina NovaSeq 6000 (Homo sapiens) was used to generate these data. GSE163154 are microarray datasets of carotid atherosclerotic plaques included non‐IPH group & IPH group.^16^ GSE120521 consists of an RNA‐seq profile of 4 stable and 4 unstable plaque samples,^17^ which was tested on the GPL16791 platform. R software (version: 4.0.1) was used for all the analyses in the manuscript.

### 2.2 Single‐cell RNA seq analysis

The R package Seurat (version 3.1.2) was used to perform dimensionality reduction, visualization and analysis of single‐cell RNA‐seq data. Cells that expressed <200 or >4000 genes or more than 10% of mitochondrial genes were considered abnormal and filtered out. Genes that have mean expression between 0.1 and 10 and dispersion between 1.25 and 20 were selected as highly variable genes. To remove batch effect across samples, we performed the FindIntegrationAnchors analysis built‐in Seurat package. Principal component analysis (PCA) was performed, and the number of the significant principal components was calculated using the built‐in ‘ElbowPlot’ function. The non‐linear dimensional reduction was realized by t‐SNE. Cluster biomarkers were found by using CellMarker. Marker genes in scRNA‐seq profiles with a minimum log‐fold change threshold of 0.25 and with *p* values computed with a Wilcoxon rank‐sum test were calculated by the ‘FindAllMarkers’ function in the Seurat package.

### 2.3 Identification of differentially expressed genes

For scRNA‐seq data, the DEGs between different cell clusters of GSE155512 were performed by MAST package in R software. DEGs with adjusted *p* values <0.05 were defined significant, whereas the up‐regulated DEGs were considered if the average logFC >0 and average logFC <0 for down‐regulated DEGs. For bulk transcriptome data including GSE120521 and GSE163154, the DEGs between two clusters were determined using the limma package in R. T‐test method was used to calculate the *p*‐value, and the adjusted *p*‐value for multiple testing was calculated using the Benjamini–Hochberg correction. We set the principal standards of | log (fold change) | >1 and *p* < 0.05 to acquire DEGs that are significant from the dataset GSE120521. The criteria for GSE163154 were as follows: the adjusted *p* < 0.05 and | log FC | >1.

### 2.4 Functional enrichment analyses

Gene ontology (GO) is an international standardized gene functional classification system and is used to describe the roles of genes and gene products in any organism based on existing biological knowledge. The Kyoto Encyclopedia of Genes and Genomes (KEGG) database is the major public pathway‐related database for analysing metabolic pathways and gene signalling networks. We used the DAVID website for gene annotation and visualization to perform the GO‐BP and KEGG pathway analyses. The functional category with a *p*‐value <0.05 was considered significant. The results were visualized using the R package, including ggplot2 and barplot.

### 2.5 PPI network construction

The protein–protein interaction (PPI) network of DEGs (GSE155512) was built through the STRING (version 11.0) website to explore the interrelationship of DEG encoded proteins. The whole networks were constructed using Cytoscape software and analysed with its Molecular Complex Detection (MCODE), which is a well‐known automated method for detecting highly interconnected subgraphs as molecular complexes or clusters in large PPI networks.

### 2.6 Haematoxylin and eosin staining

Human atherosclerotic carotid arteries were obtained from symptomatic patients undergoing endarterectomy as part of a clinical research protocol approved by the First Affiliated Hospital of USTC (University of Science and Technology of China). All patients were fully informed of the research and signed the informed consent forms. Human carotid arteries were fixed with 10% formalin before paraffin embedding. A Hematoxylin and Eosin Staining Kit (C0105, Beyotime) was used for haematoxylin and eosin (H&E) staining according to the manufacturer's protocol.

### 2.7 Immunohistochemistry

Serial sections of human atherosclerotic carotid arteries were also used for immunohistochemistry. Briefly, the sections of human carotid arteries were deparaffinized and rehydrated. After antigen retrieval with citrate buffer (P0088, Beyotime), the sections were incubated with an anti‐human AOC3 antibody (1:200, 14365‐1‐AP, Proteintech) or a rabbit IgG control at 4°C overnight followed by incubation with an HRP‐conjugated secondary antibody (1 μg/ml, GK500705, Genetech) for 30 min. The sections were stained using a DAB solution. Images were analysed using a microscope (LEICA DM2000) equipped with a camera and cellSens software (Olympus).

## 3 RESULTS

### 3.1 Single‐cell profiles of symptomatic or asymptomatic human carotid artery plaque

**FIGURE 1:**
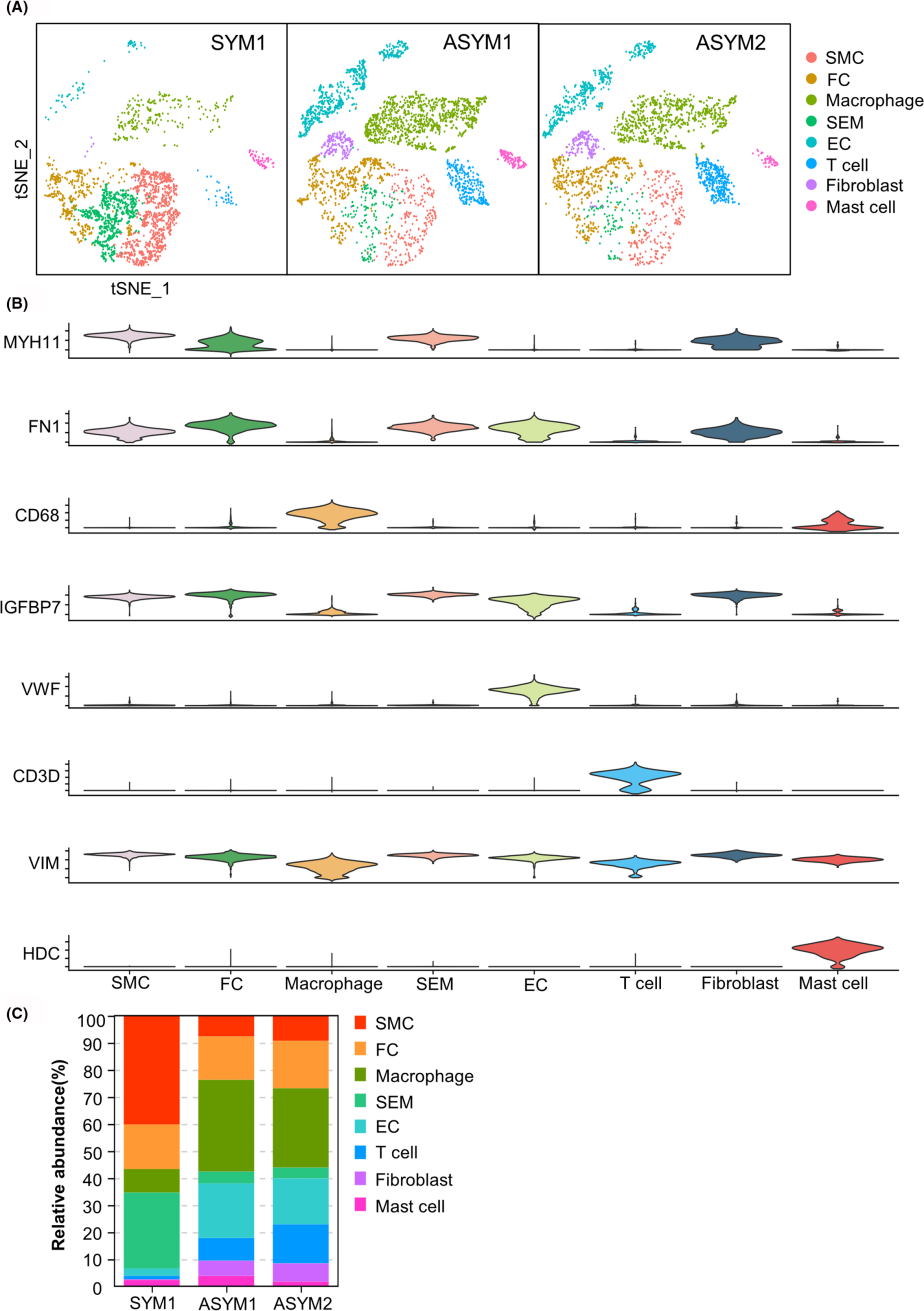
Single‐cell RNA‐sequencing atlas of symptomatic or asymptomatic human carotid artery plaque. (A) T‐Distributed Stochastic Neighbor Embedding (t‐SNE) represents the aligned gene expression data in single cells extracted from symptomatic carotid artery plaque (SYM1) or asymptomatic carotid artery plaques (ASYM1 and ASYM2). (B) The stacked violin plots showing the expression of known cell markers in each cell cluster. (C) Stacked bar chart showing the relative abundance of the eight major cell types identified by scRNA‐seq of symptomatic or asymptomatic human carotid artery plaques. ASYM, asymptomatic carotid artery plaque; EC, endothelial cells; FC, fibrochondrocytes; SEM, SMC‐derived intermediate cells; SMC, vascular smooth muscle cells; SYM, symptomatic carotid artery plaque

To reveal the heterogeneity of human atherosclerotic carotid arteries between symptomatic patients and asymptomatic patients, we analysed the original single‐cell RNA data collected from the GEO database. Using 2000 variable genes with similar profiles, unsupervised Seurat‐based clustering showed the single‐cell distributions of symptomatic carotid artery plaque (SYM1) and asymptomatic carotid artery plaques (ASYM1 and ASYM2) by t‐SNE visualizations. Mainly eight cell types, including vascular smooth muscle cells (SMCs), fibrochondrocytes (FCs), macrophages, SMC‐derived intermediate cells (SEMs), endothelial cells (ECs), T cells, fibroblasts and mast cells, were identified from the different carotid artery plaques (Figure 1A).

The marker genes that had been reported in mouse tissues with high‐quality specificity were selected for cell‐type identification of atherosclerotic carotid artery. The stacked violin plots showing the expression of known cell markers in each defined cell cluster. For example, the SMC‐specific marker gene MYH11 was highly expressed on the related cluster and therefore verified this cell cluster as SMCs (Figure 1B). In order to reveal the differences between the two groups, we computed the relative abundance of the eight main cell types in symptomatic or asymptomatic human carotid plaques identified by scRNA‐seq (Figure 1C). Interestingly, the relative abundance of ECs in asymptomatic arterial plaques was obviously increased compared to symptomatic arterial plaque. On the contrary, SMCs were relatively reduced in asymptomatic arterial plaques.

### 3.2 Distinct gene expression profiles of three endothelial cell subpopulations

**FIGURE 2:**
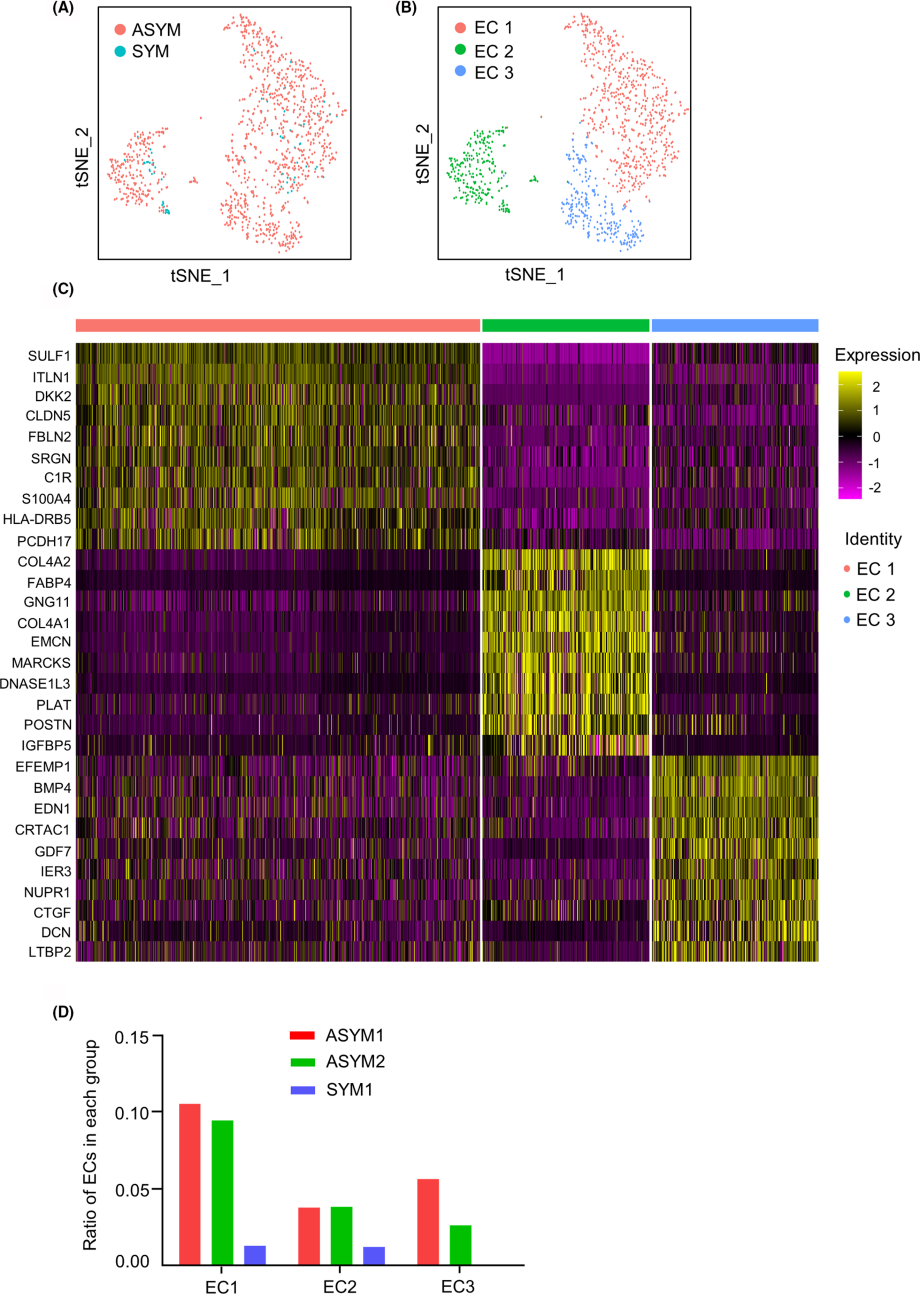
Distinct endothelial cell subpopulations and their gene expression signatures. (A) t‐SNE plots of endothelial cell subpopulations from the SYM (*n* = 1) or ASYM (*n* = 2) group. (B) t‐SNE plot of three distinct EC subclusters from SYM and ASYM. (C) Heatmap showing the 10 most up‐regulated genes in each defined EC subcluster. (D) The ratio of the number of endothelial cells in the three distinct EC subclusters to the total cells in each group. ASYM, asymptomatic carotid artery plaque; EC, endothelial cells; SYM, symptomatic carotid artery plaque

To uncover the EC heterogeneity between symptomatic and asymptomatic human carotid plaques. We re‐clustered endothelial cells detected from SYM and ASYM groups (Figure 2A). These cells were clustered into three distinct subpopulations, including EC 1, EC 2 and EC 3 (Figure 2B). To show the distinct gene expression profiles among three EC subpopulations, we calculated the cell markers of each EC subpopulation and showed the expression of the top 10 significantly enriched genes (Figure 2C). The ratio of the number of endothelial cells in the three distinct EC subclusters to the total cells in each sample was displayed by the histogram (Figure 2D). EC 1 and EC 2 presented in both ASYM and SYM groups with the vast majority of cells in the ASYM group, while EC 3 was mostly from the ASYM group, indicating a difference of cellular heterogeneity in carotid plaques between symptomatic and asymptomatic patients.

### 3.3 Functional enrichment analysis of biological processes in three distinct EC subpopulations

**FIGURE 3:**
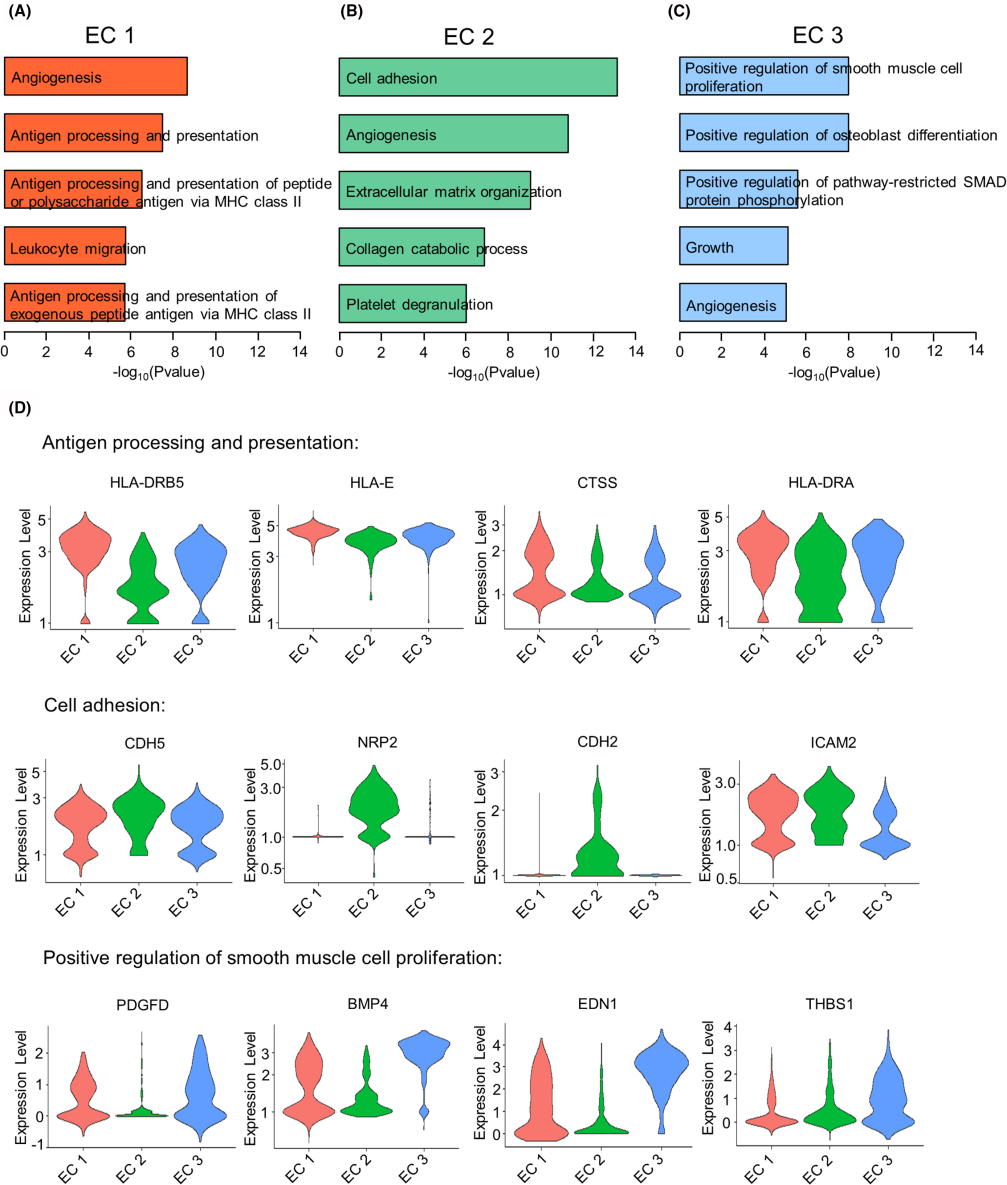
Gene ontology enrichment analysis of biological processes in three distinct EC subpopulations. (A) Bar plot represents the top five enriched biological processes in EC 1 subcluster (red). (B) Bar plot represents the top five enriched biological processes in EC 2 subcluster (green). (C) Bar plot represents the top five enriched biological processes in EC 3 subcluster (blue). (D) Violin plots of antigen processing and presentation‐associated genes (HLA‐DRB5, HLA‐E, CTSS and HLA‐DRA), cell adhesion‐associated genes (CDH5, NRP2, CDH2 and ICAM2) and positive regulation of smooth muscle cell proliferation‐associated genes (PDGFD, BMP4, EDN1 and THBS1) expression in all three EC subclusters. EC, endothelial cells

To investigate the functional heterogeneity of each EC subpopulation, gene ontology (GO) enrichment analysis of biological processes was performed to analyse each cell cluster. Interestingly, EC 1 had a particular set of functions such as angiogenesis, antigen processing and presentation, and leukocyte migration, which is consistent with the immune response (Figure 3A). EC 2 plays a vital role in cell adhesion, angiogenesis, extracellular matrix organization and collagen catabolic process (Figure 3B). Compared to EC 1 and EC 2, the genes involved in the positive regulation of smooth muscle cell proliferation, osteoblast differentiation and pathway restricted SMAD protein phosphorylation were highly expressed on EC 3 subpopulation from the ASYM group (Figure 3C) while EC 3 was not detectable in the SYM group (Figure 2A,D).

Furthermore, we found that HLA‐DRB5, HLA‐E, CTSS and HLA‐DRA genes that encode molecules involved in antigen processing and presentation were up‐regulated in EC 1 (Figure 3D). Moreover, compared with other clusters, genes regulating cell adhesion, such as CDH5, NRP2, CDH2 and ICAM2, were selectively and highly expressed in EC 2 (Figure 3D). Genes associated with positive regulation of smooth muscle cell proliferation (PDGFD, BMP4, EDN1 and THBS1) were highly expressed in EC 3 (Figure 3D). Together, three distinct EC subpopulations with specific biological processes were detected on human carotid plaques.

### 3.4 Differential gene expression profile of EC 1 subcluster between ASYM and SYM

**FIGURE 4:**
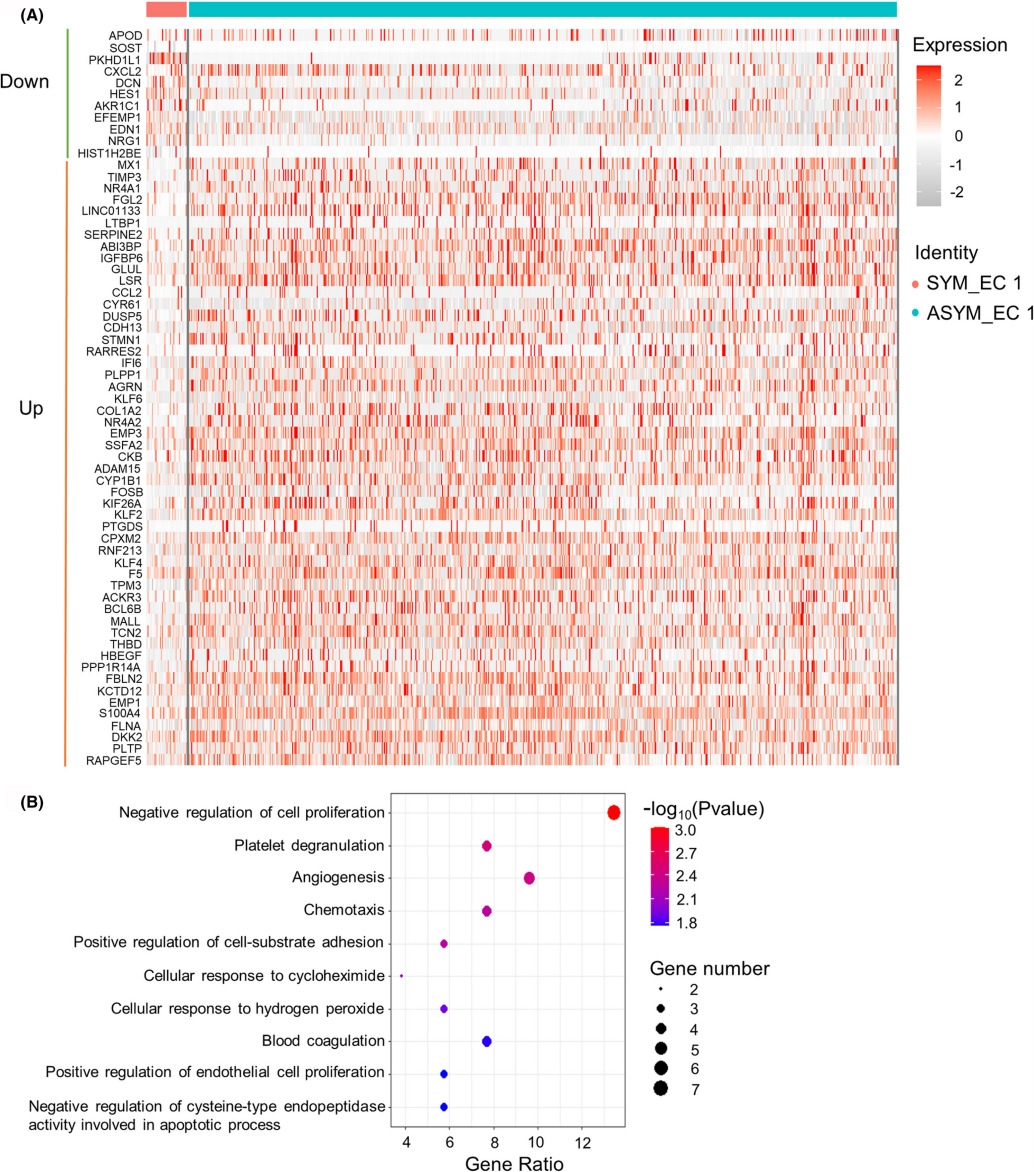
Differential gene expression signature between ASYM and SYM in EC 1 subcluster. (A) Heatmap showing the differentially expressed genes between asymptomatic and symptomatic carotid plaques in the EC 1 subpopulation. (B) Bubble plot showing the top 10 enriched biological processes in EC 1 subcluster from ASYM group. ASYM, asymptomatic carotid artery plaque; EC, endothelial cells; SYM, symptomatic carotid artery plaque

To observe the differences between the endothelial cells derived from asymptomatic and symptomatic carotid plaques in the EC 1 subpopulation, we calculated the differentially expressed genes between ASYM and SYM groups using the MAST package. The heatmap showed the expression of differential genes of the ASYM and SYM groups (Figure 4A). The colour represents the expression level for each gene. Red represents high expression and grey represents low expression. Eleven genes were down‐regulated while 52 genes were up‐regulated in the AYSM group.

We next assessed the potential function of the EC 1 subcluster in ASYM group compared to the SYM group using 52 differentially expressed genes. Top 10 enriched biological processes were found based on the *p*‐value, of which five were negative regulation of cell proliferation, platelet degranulation, angiogenesis, chemotaxis and positive regulation of cell‐substrate adhesion (Figure 4B). These results indicated that the biological processes involved in cell proliferation, platelet activation and angiogenesis are essential mediators in asymptomatic carotid plaques.

### 3.5 Specific functions of EC 2 subpopulation in ASYM group

**FIGURE 5:**
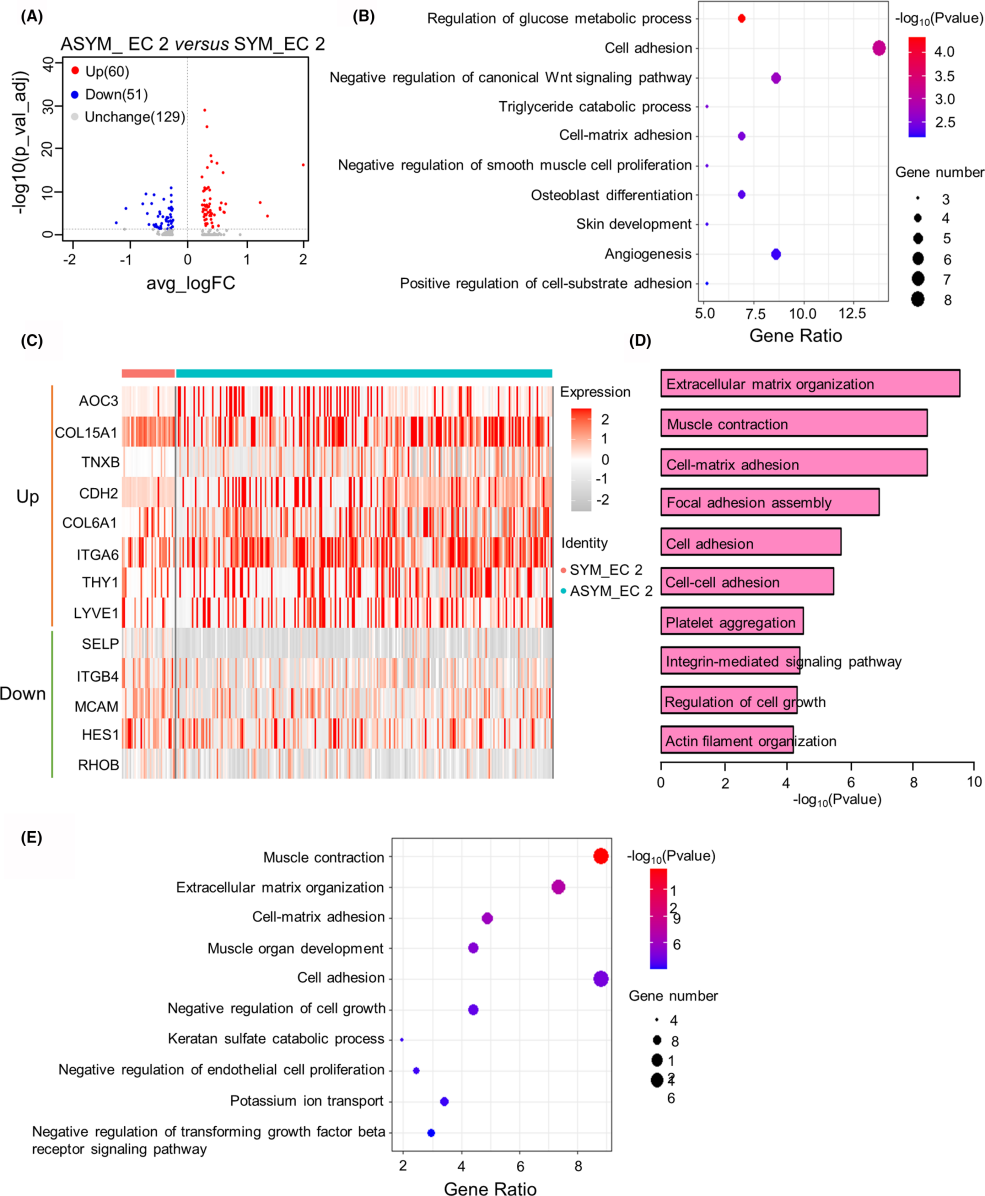
Specific functions of EC 2 subcluster in ASYM group. (A) Volcano plot showing the differentially expressed genes between asymptomatic and symptomatic carotid plaques in the EC 2 subpopulation. Genes in red are considered up‐regulated in the ASYM group while genes in blue are considered up‐regulated in the SYM group. (B) Bubble plot showing the top 10 enriched biological processes up‐regulated in EC 2 subcluster from ASYM group. (C) Heatmap showing the cell adhesion‐associated genes expressed in EC 2 subcluster of ASYM and SYM groups. (D) The bar plot represents the top ten enriched biological processes in stable plaque compared to unstable plaque. (E) Bubble plot showing the top 10 enriched biological processes in the non‐IPH group compared to the IPH group. ASYM, asymptomatic carotid artery plaque; EC, endothelial cells; IPH, intraplaque haemorrhage; SYM, symptomatic carotid artery plaque

To elucidate the specific role of EC 2 subpopulation in asymptomatic carotid artery plaques, we identified 240 differentially expressed genes (DEGs) in the EC 2 subpopulation of ASYM group compared to the SYM group using the MAST package (Figure 5A). Among them, 60 DEGs were up‐regulated (adjusted *p*‐value <0.05 and avg logFC >0), and the remaining 51 DEGs were down‐regulated (adjusted *p*‐value <0.05 and avg logFC <0) in the ASYM group compare to the SYM group (Figure 5A). The bubble plot showed the top 10 enriched up‐regulated biological processes in EC 2 subcluster of the ASYM group, such as regulation of glucose metabolic process, cell adhesion and negative regulation of canonical Wnt signalling pathway (Figure 5B). Moreover, the expression of cell adhesion‐related genes in EC 2 subcluster from the ASYM and SYM groups was shown by heatmap (Figure 5C).

We found that AOC3, COL15A1, TNXB, CDH2 and COL6A1 were up‐regulated in the plaques from the ASYM group compared to the SYM group. Due to the symptomatic plaques tend to be unstable and prone to rupture, we utilized the GEO datasets with the accession numbers GSE120521 and GSE163154 to further validate the similarities of biological process in the development of carotid atherosclerotic plaques. GSE120521 consisted of an RNA‐seq profile of 4 stable and 4 unstable human carotid plaques obtained at carotid endarterectomy in symptomatic patients. The bar plot represented the top 10 enriched biological processes in stable plaques compared to unstable plaques (Figure 5D). Of note, the biological process of cell adhesion was also found in the 5th top‐enriched biological processes of sable plaques. In order to determine the role of cell adhesion in intraplaque haemorrhage, GSE163154 dataset which includes the non‐IPH (intraplaque haemorrhage) and IPH group was employed. After differentially expressed genes were calculated, we used a bubble plot to show the top 10 enriched biological processes in the non‐IPH group (Figure 5E). Surprisingly, we noticed that genes that encode molecules involved in cell adhesion and cell‐matrix adhesion were up‐regulated in non‐IPH carotid plaques. Together, the integrated analysis of single‐cell RNA‐seq and bulk transcriptome data revealed that the function of cell adhesion may play an important role in clinical events such as plaque stability and intraplaque haemorrhage.

### 3.6 **AOC3** **may be related to plaque haemorrhage and plaque stability**

**FIGURE 6:**
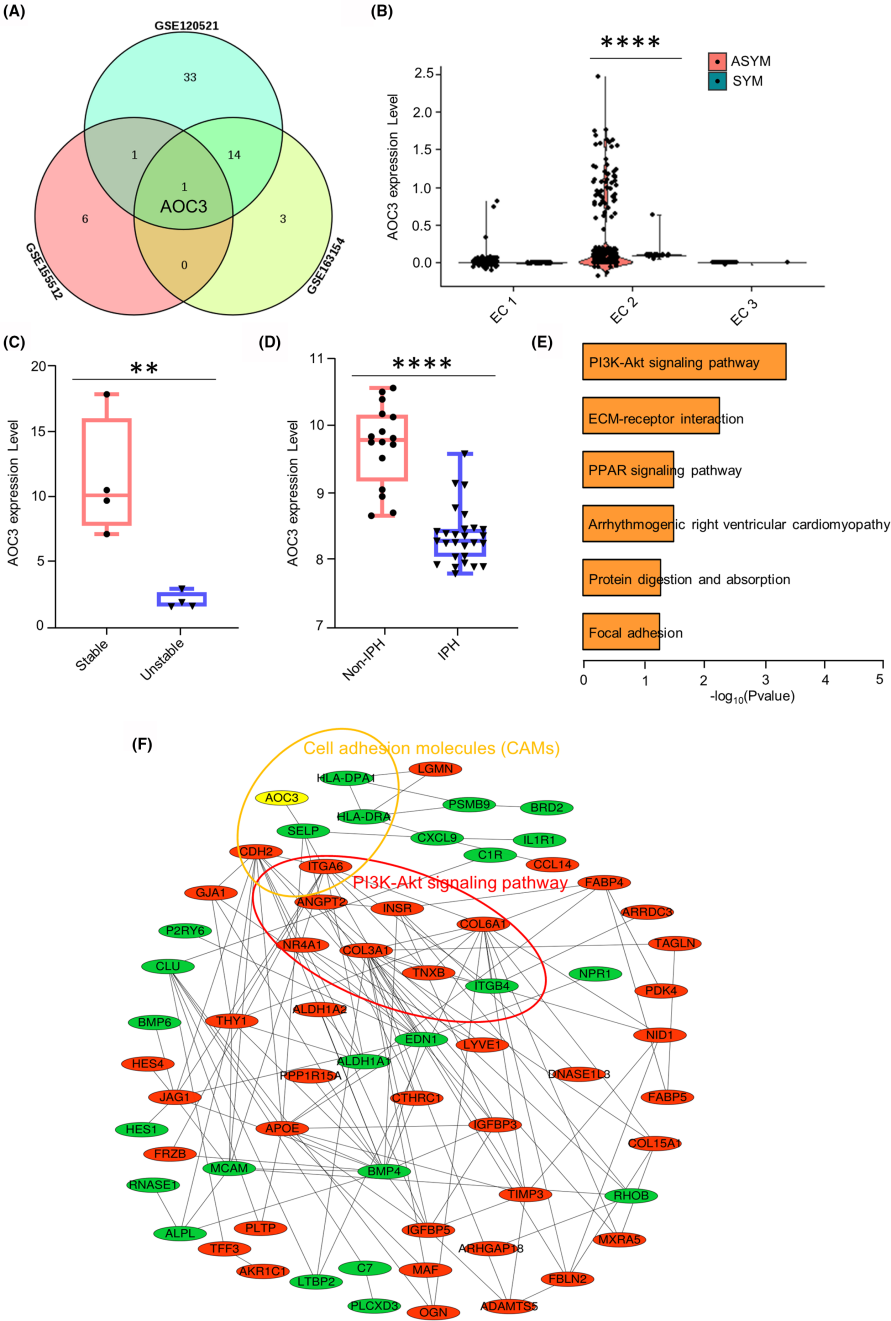
AOC3 is related to plaque haemorrhage and plaque stability. (A) Venn plot showing the overlapping relationships of up‐regulated genes about cell adhesion in ASYM, stable and non‐IPH group. (B) Violin plots of AOC3 expression in all three EC subpopulations. (C) The AOC3 expression in stable plaques (*n* = 4) and unstable plaques (*n* = 4). (D) The AOC3 expression in non‐IPH (*n* = 16) and IPH (*n* = 27) group. (E) Gene ontology enrichment analysis of KEGG pathway with up‐regulated genes in EC 2 subcluster of ASYM group. (F) A protein interaction network was constructed using all differentially expressed genes in the EC 2 subcluster. Red colour represents up‐regulated genes, and green colour represents down‐regulated genes. ASYM, asymptomatic carotid artery plaque; EC, endothelial cells; IPH, intraplaque haemorrhage

To investigate the role of cell adhesion‐related genes in atherosclerotic plaque development, the gene expression profiles of GSE155512, GSE120521 and GSE163154 were integrated. We applied the Venn diagram to identify the overlapping relationships of up‐regulated genes about cell adhesion among ASYM, stable and non‐IPH groups (Figure 6A). Of note, AOC3 is the only intersection gene related to plaque haemorrhage, plaque stability and asymptomatic plaque. Furthermore, the violin plot was used to analyse AOC3 expression in all three EC subpopulations of symptomatic carotid artery plaque and asymptomatic carotid artery plaques (Figure 6B). We found that AOC3 was highly expressed in EC 2 subcluster of the ASYM group. Moreover, the expression of AOC3 in stable plaques and non‐IPH plaques is significantly up‐regulated (Figure 6C,D).

To further investigate the underlying mechanism of the up‐regulated genes from EC 2 subcluster in the ASYM group, Gene ontology enrichment analysis of the KEGG pathway was performed utilizing the DAVID online tool (Figure 6E). The top 5 enriched entries for KEGG pathways were PI3K‐Akt signalling pathway, ECM‐receptor interaction, PPAR signalling pathway, arrhythmogenic right ventricular cardiomyopathy and focal adhesion. Since most genes of the ASYM group are involved in the PI3K‐Akt signalling pathway, we proposed that AOC3 genes may be regulated by the PI3K‐Akt signalling pathway. To test this hypothesis, we constructed a protein interaction network using all differentially expressed genes in the EC 2 subpopulation (Figure 6F). Red colour represents up‐regulated genes, while green colour represents down‐regulated genes. Results showed that the expression of the AOC3 gene may be regulated by ITGA6 that participated in both PI3K‐Akt signalling and cell adhesion pathways.

**FIGURE 7:**
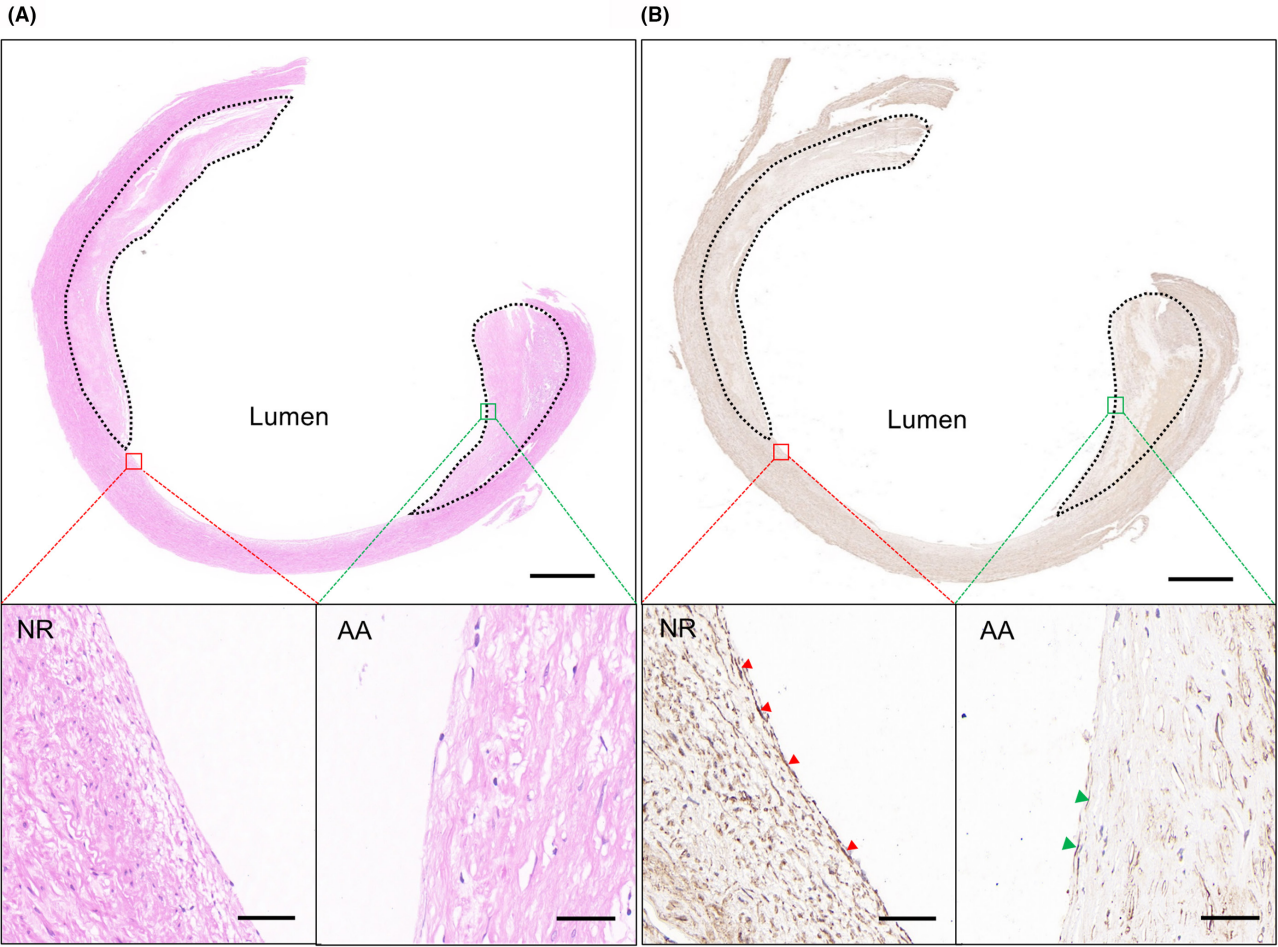
AOC3 expression in carotid atherosclerotic plaques from symptomatic patients. (A) carotid intima sections were stained with haematoxylin–eosin (bar = 1000 µm). The magnified images of the atherosclerotic area (AA) and non‐plaque region (NR) are shown below (bar = 50 µm). (B) Representative immunohistochemical staining images for AOC3 in AA and NR regions. The dotted lines define the regions of the plaques. Nuclei were stained by haematoxylin (bar = 1000 µm). The magnified image was shown below, where the arrows indicated the AOC3 expression in nucleated cells of the intima (bar = 50 µm)

To examine the expression of endothelial AOC3 in atherosclerotic plaques of symptomatic patients undergoing carotid endarterectomy, we immunostained on carotid intima tissue sections of atherosclerotic plaques of carotid arteries in symptomatic patients. We first defined the area of both the atherosclerotic area (AA) and non‐plaque region (NR) in the carotid intima by haematoxylin–eosin staining (Figure 7A) and then examined the expression of AOC3 within the plaque. Results showed that AOC3 was expressed in the atherosclerotic area of carotid intima, but appeared less abundant compared to the non‐plaque region of intima (Figure 7B). Thus, these data support the role of AOC3 in plaque haemorrhage and plaque stability.

## 4 DISCUSSION

In this study, we employed the single‐cell RNA‐seq data of human carotid artery plaques in symptomatic or asymptomatic patients. Three distinct EC subpopulations were identified by Seurat‐base‐unsupervised clusters. GO enrichment analysis revealed that the EC 1 subpopulation was endowed with antigen processing and presentation and that EC 2 had a specific function in cell adhesion, while EC 3 expressed most genes involved in smooth muscle cell proliferation. Interestingly, the EC 2 subpopulation of the ASYM group also showed an explicit function of cell adhesion comparing the SYM group. Furthermore, the integrated analysis revealed that the 5th top‐enriched biological process was cell adhesion in the stable or non‐haemorrhaged plaques compared to unstable plaques or haemorrhaged plaques. Finally, cell adhesion‐related gene AOC3 was co‐expressed on asymptomatic plaques, stable plaques, or non‐haemorrhaged plaques and may be regulated by the PI3K‐Akt signalling pathway through ITGA6.

Endothelial cell heterogeneity has largely improved our understanding of distinct EC functions in cardiovascular disease. Normal ECs of a healthy vessel wall maintain homeostasis through metabolism, vasodilation, anticoagulation and anti‐inflammation.^18^ However, ECs will have unique features and exhibit different phenotypes when the pathological process occurs. EC subpopulations with different phenotypes including proliferative ECs, inflammatory ECs, remodelling ECs, ECs involved in endothelial‐mesenchymal transition (EndMT) and ECs involved in angiogenesis were identified under different stimuli such as oxidative stress, vascular injury and disturbed flow.^19^ Importantly, EC subpopulation involved in angiogenesis was reported in normal mouse aortic cells using single‐cell RNA‐sequencing,^11^ which is consistent with the top 1 biological process of EC 1 we detected. EC 3 subpopulation was more specialized in the proliferation of smooth muscle cells and osteoblast differentiation possibly in line with mesenchymal‐like cells that undergo a phenotypic switch towards a mesenchymal phenotype.^8^, ^20^ However, whether the functions of EC 2 subpopulation were related to inflammatory ECs still needs further research.

Amine oxidase copper containing 3 (AOC3), also known as VAP‐1 (vascular adhesion protein 1), is a member of the semicarbazide‐sensitive amine oxidase family. The human AOC3 gene is located on chromosome 17, and it is expressed on smooth muscle cells/pericytes, adipocytes and especially endothelial cells^21^ as a transmembrane glycoprotein with a molecular weight of 170 ~ 180 kDa.^22^ Besides the transmembrane form, soluble AOC3 protein has been detected in the circulation and mainly released by vascular endothelial cells.^23^ For the biological function, Aalto et al. showed that increased soluble AOC3 concentrations may have a deleterious influence on endothelial integrity.^24^ AOC3 is the most important source of primary amine oxidase in human serum and has a role in the leukocyte‐endothelial cell interactions,^25^, ^26^ adipocyte differentiation,^27^ glucose transport^28^ and structural organization of vascular smooth muscle. The combination of oxidase activity and adhesion capacities as well as its strong association with inflammatory pathologies makes AOC3 an interesting therapeutic target for multiple human diseases.

The high activity level of AOC3 has been reported to participate in the development of type 2 diabetes mellitus, Alzheimer's disease, atherosclerosis and stroke.^29^ Especially, studies have found that AOC3 is highly expressed in the atherosclerotic plaques of human carotid arteries^30^ and the aorta of hypercholesterolemia rabbits.^31^ Endothelial AOC3 could recruit monocytes and Th1 CD4+ cells into the vascular wall to promote the formation of atherosclerotic lesions.^32^ Of note, the level of soluble AOC3 is associated with risk factors for atherosclerosis, carotid artery intimal thickening and clinical cardiovascular events in asymptomatic individuals,^33^ which is consistent with our scRNA‐seq results. Compared with symptomatic carotid atherosclerotic plaques, AOC3 expression is significantly up‐regulated in the EC 2 subpopulation of asymptomatic carotid atherosclerotic plaques. Furthermore, we also found that the expression of AOC3 in stable plaques and non‐IPH plaques is significantly up‐regulated by integrating the original data from different patients with atherosclerosis. However, it is difficult to conclude that whether AOC3 plays a protective or destructive role in the entire development of atherosclerosis. We only found that AOC3 was specifically and highly expressed in the EC 2 subcluster of the ASYM group, suggesting that endothelial cell AOC3 plays an important role in the formation of asymptomatic plaques. However, whether there is a difference in AOC3 expression between symptomatic plaques and asymptomatic plaques of other cell types, such as smooth muscle cells and macrophages, is worthy of further investigation.

Several limitations should be noticed in our study. First, our single‐cell RNA‐seq data of human carotid artery plaques were obtained only from one symptomatic and two asymptomatic patients. As the sample size increases, the proportions of the three EC subpopulations will be better counted. Second, our results revealed the differences in symptomatic and asymptomatic carotid plaques based on the transcriptome, but further confirmation is warranted in proteomics. Third, bioinformatics analysis indicated that the function of cell adhesion plays a vital role in different stages of the development of atherosclerosis. However, more biological experiments are needed to verify this result.

In summary, we identified three distinct EC subpopulations in symptomatic and asymptomatic human carotid artery plaques. GO enrichment analysis revealed different biological processes among these EC subpopulations such as antigen processing and presentation, cell adhesion, and smooth muscle cell proliferation. Of note, the differentially expressed genes of the EC 2 subpopulation showed that the genes involved in cell adhesion were up‐regulated in asymptomatic plaques compared to symptomatic plaques. Consistently, integrating the data of intraplaque haemorrhage and plaque stability implied that the biological function of cell adhesion was up‐regulated in sable and non‐intraplaque haemorrhage plaques. Targeting cell adhesion and the specialized genes may provide potential new therapeutic directions to prevent asymptomatic patients from stroke.

## AUTHOR CONTRIBUTIONS

**Fengchan Li:** Investigation (equal); Methodology (equal); Writing – original draft (equal). **Yun Du**: Methodology (equal); Writing – original draft (equal). **Lei Hong:** Investigation (equal); Methodology (equal). **Ziting Liu:** Investigation (equal). **Kunmin Yan:** Methodology (supporting). **Chu Liu:** Methodology (supporting). **Zhen Zhu:** Investigation (supporting). **Qiongyu Lu:** Investigation (equal). **Chaojun Tang:** Formal analysis (equal); Writing – original draft (equal). **Li Zhu:** Investigation (equal); Methodology (equal); Supervision (equal); Writing – original draft (equal); Writing – review & editing (equal).

## CONFLICT OF INTEREST

The authors have declared that no competing interest exists.
